# Supplementary material for: Human Cytomegalovirus-Encoded G Protein-Coupled Receptor UL33 Facilitates Virus Dissemination via the Extracellular and Cell-to-Cell Route
Source: Viruses. 2020 May 30;12(6):594. doi: 10.3390/v12060594 (PMC7354556; doi:10.3390/v12060594)
Supplement: Supplementary file 1 [file viruses-12-00594-s001.pdf]

Supplementary Figures

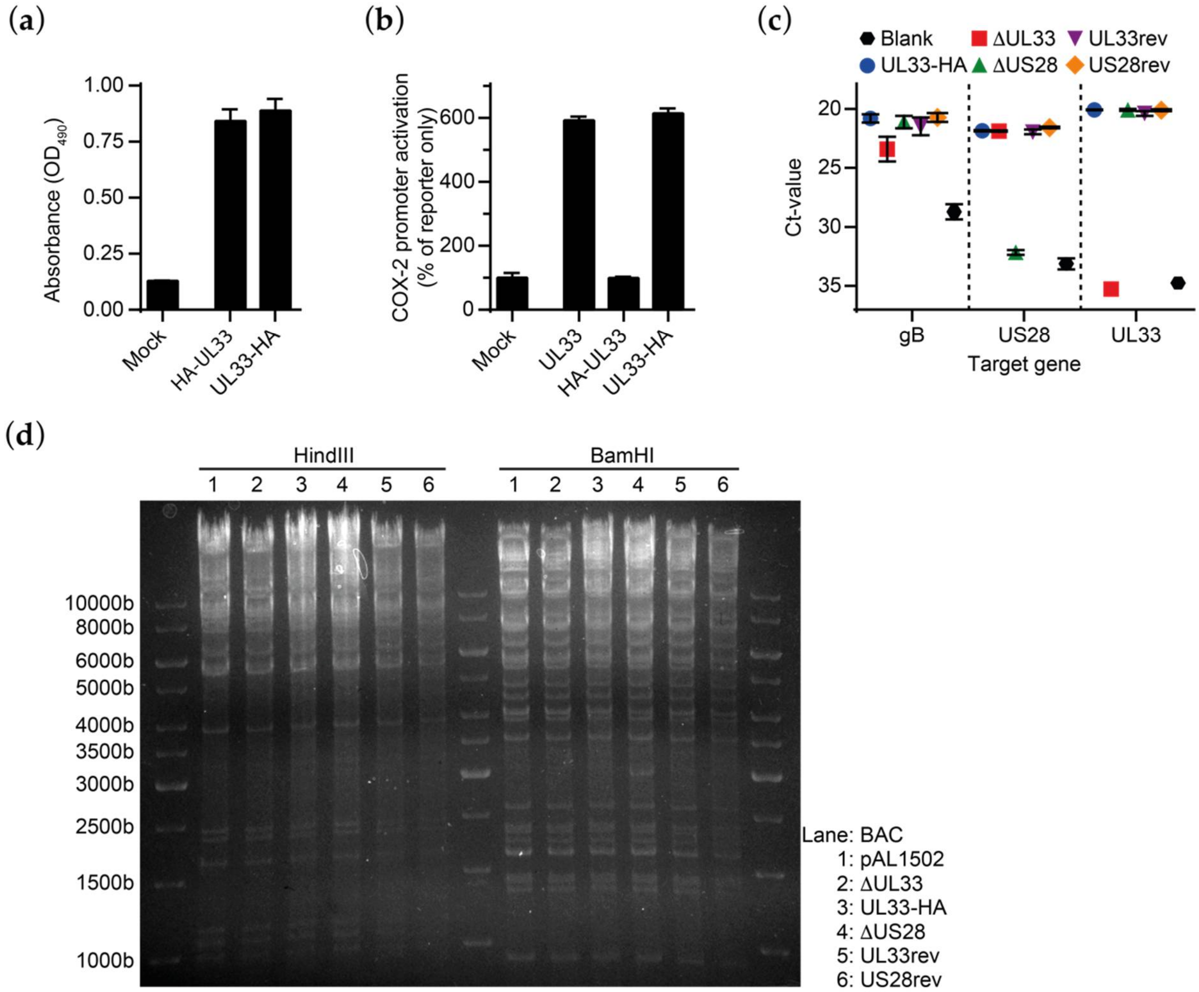

Figure S1. Validation of recombineering strategy and genomic integrity of HCMV Merlin BAC variants. Functionality check of N- or C-terminally HA-tagged UL33 receptors. HEK293T cells were transfected with receptor DNA and/or COX-2 promoter-driven reporter gene. The next day, receptor expression (**a**) and receptor signaling (**b**) were determined. Quality control of HCMV Merlin BAC derivatives as assessed by qRT-PCR of US28 and UL33 genes (**c**) and HindIII and BamHI endonuclease digestion pattern analysis (d).

```
Strain          UL33 receptor amino acid sequence                                Residue

Merlin          MDTIIHNTTNRSTDTPHVNITCNITEPLSAIRTTEAVINTFIIFVGGPLNAIVLITQLLT     60
Towne           MDTIIHNTTNRSTSTPHVNSTCNMTETLSAIRTTEAVINTFIIFVGGPLNAIVLVTQLLT     60
Toledo          MDTIIHNST-RNTTTPHINDTCNMTGPLFAIRTTEAVLNTLIIFVGGPLNAIVLVTQLLT     59
TB40/E          MDTIIHNST-RNNTPPHINDTCNMTGPLFAIRTTEAVLNTFIIFVGGPLNAIVLITQLLT     59
AD169           MDTIIHNST-RNNTPPHINDTCNMTGPLFAIRTTEAVLNTFIIFVGGPLNAIVLITQLLT     59
FIX/PH/TR       MDTIIHNST-RNNTPPHINDTCNMTGPLFAIRTTEAVLNTFIIFVGGPLNAIVLITQLLT     59
                *******:* *..  **:* ***:*  * ********:**:*************:*****

Merlin          NRVLGYSTPTIYMTNLYSTNFLTLTVLPFIVLSNQWLLPASVASCKFLSVIYYSSCTVGF     120
Towne           NRVLG STPTIYMTNLYSTNFLTLTVLPFIVLSNQWLLPASVTSCKFLSVIYYSSCTVGF     120
Toledo          NRVLGYSTPTIYMTNLYSTNFLTLTVLPFIVLSNQWLLPAGVASCKFLSVIYYSSCTVGF     119
TB40/E          NRVLGYSTPTIYMTNLYSTNFLTLTVLPFIVLSNQWLLPAGVASCKFLSVIYYSSCTVGF     119
AD169           NRVLGYSTPTIYMTNLYSTNFLTLTVLPFIVLSNQWLLPAGVASCKFLSVIYYSSCTVGF     119
FIX/PH/TR       NRVLGYSTPTIYMTNLYSTNFLTLTVLPFIVLSNQWLLPAGVASCKFLSVIYYSSCTVGF     119
                ****************************************.*:*****************

Merlin          ATVALIAADRYRVLHKRTYARQSYRSTYIILLLTWFAGLIFSMPAAVYTTVVIHNGTNG-     179
Towne           ATVALIAADRYRVLHKRTYARQSYRSTYIILLLTWFAGLIFSMPAAVYTTVVIHNGTDE-     179
Toledo          ATVALIAADRYRVLHKRIYARQSYRSTYTILILTWLAGLIFSMPAAVYTTVVMHHEANDT     179
TB40/E          ATVALIAADRYRVLHKRTYARQSYRSTYMILLLTWLAGLIFSVPAAVYTTVVMHHDANDT     179
AD169           ATVALIAADRYRVLHKRTYARQSYRSTYMILLLTWLAGLIFSVPAAVYTTVVMHHDANDT     179
FIX/PH/TR       ATVALIAADRYRVLHKRTYARQSYRSTYMILLLTWLAGLIFSVPAAVYTTVVMHHDANDT     179
                ***************** ********** **:***:******:*********:*: ::

Merlin          QSSNGHATCVLYFIADEVYTVLLSWKVLLTLVWGAAPVIMMTWFYAFFYSTVQRASQKQR     239
Towne           -NTNGHATCVLYFIADEVYTVLLSWKVLLTLVWGAAPVIMMTWFYAFFYSTVQRASQKQR     238
Toledo          TNATGHATCVLYFVADEVHTVLLSWKVLLTLVWGAAPVIMMTWFYAFFYSTVQRTSQKQR     239
TB40/E          NNTNGHATCVLYFVAEEVHTVLLSWKVLLTLVWGAAPVIMMTWFYAFFYSTVQRTSQKQR     239
AD169           NNTNGHATCVLYFVAEEVHTVLLSWKVLLTMVWGAAPVIMMTWFYAFFYSTVQRTSQKQR     239
FIX/PH/TR       NNTNGHATCVLYFVAEEVHTVLLSWKVLLTLVWGAAPVIMMTWFYAFFYSTVQRTSQKQR     239
                 .:.*********:*:**:***********:***********************:*****

Merlin          SRTLTFVSVLLISFVALQTPYVSIMIFNSYATAAWPMDCEHLTLRRTIGTLSRLVPHLHC     299
Towne           SRTLTFVSVLLISFVALQTPYVSIMIFNSYATAAWPMDCEHLTLRRTIGTLSRLVPHLHC     298
Toledo          SRTLTFVSVLLISFVALQTPYISLMIFNSYATTAWSTKCEHLTLRRTIGTLARLVPHLHC     299
TB40/E          SRTLTFVSVLLISFVALQTPYVSLMIFNSYATTAWPMQCEHLTLRRTIGTLARVVPHLHC     299
AD169           SRTLTFVSVLLISFVALQTPYVSLMIFNSYATTAWPMQCEHLTLRRTIGTLARVVPHLHC     299
FIX/PH/TR       SRTLTFVSVLLISFVALQTPYVSLMIFNSYATTAWPMQCEHLTLRRTIGTLARVVPHLHC     299
                *********************:*:********:**  .*************:*:******

Merlin          LINPILYALLGHDFLQRMRQCFRGQLLDRRAFLRSQQNQRATAETNLAAGNNSQSVATSL     359
Towne           LINPILYALLGHDFLQRMRQCFRGQLLDRRAFLRSQQNQRATAETNLAAGNNSQSVATSL     358
Toledo          LINPILYALLGHDFLQRMRQCFRGQLLDRRAFLRSQQNQRATAETNLAAGNNSQSVATSL     359
TB40/E          LINPILYALLGHDFLQRMRQCFRGQLLDRRAFLRSQQNQRATAETNLAAGNNSQSVATSL     359
AD169           LINPILYALLGHDFLQRMRQCFRGQLLDRRAFLRSQQNQRATAETNLAAGNNSQSVATSL     359
FIX/PH/TR       LINPILYALLGHDFLQRMRQCFRGQLLDRRAFLRSQQNQRATAETNLAAGNNSQSVATSL     359
                ************************************************************

Merlin          DTSSKNCNQHAKRSVSFNFPSGTWKGGQKTASNDTSTKIPHRLSQSHHNLSGV            412
Towne           DTNSKNCNQHAKRSVSFNFPSGTWKGGQKTASNDTSTKIPHRLSQSHHNLSGV            411
Toledo          DPNSKNCNQHAKRSVSFNFPSGTWKGGQKTASNDTSTKIPHRLSQSHHNLSGV            412
TB40/E          DTNSKNCNQHAKRSVSFNFPSGTWKGGQKTASNDTSTKIPHRLSQSHHNLSGV            412
AD169           DTNSKNYNQHAKRSVSFNFPSGTWKGGQKTASNDTSTKIPHRLSQSHHNLSGV            412
FIX/PH/TR       DPNSKNCNQHAKRSVSFNFPSGTWKGGQKTASNDTSTKIPHRLSQSHHNLSGV            412
                * .*** **********************************************
```

Figure S2. Multiple sequence alignment of HCMV UL33 orthologs. Sequence variability is highlighted in yellow. (*) fully conserved residue; (:) conservation between groups of strongly similar properties; (.) conservation between groups of weakly similar properties.
